# Circadian rhythms in rheumatology - a glucocorticoid perspective

**DOI:** 10.1186/ar4687

**Published:** 2014-11-13

**Authors:** Cornelia M Spies, Rainer H Straub, Maurizio Cutolo, Frank Buttgereit

**Affiliations:** 1 Department of Rheumatology and Clinical Immunology, Charité University Medicine Berlin, Charitéplatz 1, 10117 Berlin, Germany; 2 Laboratory of Experimental Rheumatology and Neuroendocrino-Immunology, Department of Internal Medicine I, University Hospital Regensburg, Franz-Josef-Strauss-Allee 11, 93053 Regensburg, Germany; 3 Research Laboratory and Academic Division of Clinical Rheumatology, Department of Internal Medicine, University of Genova, Viale Benedetto XV,6, 16132 Genova, Italy

## Abstract

The hypothalamic-pituitary-adrenal (HPA) axis plays an important role in regulating and controlling immune responses. Dysfunction of the HPA axis has been implicated in the pathogenesis of rheumatoid arthritis (RA) and other rheumatic diseases. The impact of glucocorticoid (GC) therapy on HPA axis function also remains a matter of concern, particularly for longer treatment duration. Knowledge of circadian rhythms and the influence of GC in rheumatology is important: on the one hand we aim for optimal treatment of the daily undulating inflammatory symptoms, for example morning stiffness and swelling; on the other, we wish to disturb the HPA axis as little as possible. This review describes circadian rhythms in RA and other chronic inflammatory diseases, dysfunction of the HPA axis in RA and other rheumatic diseases and the recent concept of the hepato-hypothalamic-pituitary-adrenal-renal axis, the problem of adrenal suppression by GC therapy and how it can be avoided, and evidence that chronotherapy with modified release prednisone effective at 02:00 a.m. can inhibit proinflammatory sequelae of nocturnal inflammation better compared with GC administration in the morning but does not increase the risk of HPA axis insufficiency in RA.

## Introduction

The circadian rhythm is generated by a central circadian oscillator in the suprachiasmatic nucleus of the hypo-thalamus [1]. This nucleus has many connections to other centers in the brain. The circadian activity of this particular nucleus is transferred to the immune system via the hypothalamic hypothalamic-pituitary-adrenal (HPA) axis, leading to the typical undulation of clinical symptoms in chronic inflammatory diseases with a maximum in the early morning hours [2]. In this review we will describe circadian rhythms in rheumatoid arthritis (RA) and other rheumatic and chronic inflammatory diseases, dysfunction of the HPA axis in RA and other rheumatic and chronic inflammatory diseases, the problem of adrenal suppression by glucocorticoid (GC) therapy, and whether or not chronotherapy with prednisone is more effective and aggravates adrenal suppression.

## Circadian rhythms in RA and other chronic inflammatory diseases

Classical symptoms of RA, such as morning stiffness and swelling, show a clear temporal relationship with nocturnally elevated levels of proinflammatory cytokines, as a consequence of a cascade of increased nocturnal inflammation [3]. Several of these cytokines, such as tumor necrosis factor (TNF) alpha and interleukin (IL)-6, are highly increased in patients with active RA in the early hours of the day, but are found at very low levels after noon [4]. Their release pattern and serum concentrations, possibly triggered by proinflammatory hormones such as melatonin (and prolactin), follow a 24-hour daily cycle [5]. Also, the cortisol rhythm - which is also present in healthy individuals, and therefore is primary, with low levels at night - may explain nocturnal inflammation. Since cortisol is the strongest endogenous anti-inflammatory substance, its downregulation during the evening and night is linked to an increase of inflammation during the early morning, and its upregulation in the early morning is most probably related to inhibition of inflammation during the day [1]. The early morning inflammatory signs, typical for many inflammatory rheumatic conditions, can thus be explained.

As noted, the proinflammatory hormones begin to rise before the daily early morning aggravation of RA symptoms, and before activation of endogenous cortisol synthesis that counteracts the cascade of the immune/ inflammatory processes [3]. In addition, rhythmic fluctuations of the nocturnal secretion and the peripheral metabolism of endogenous cortisol, as well as changes in the activation of biologically inactive to active cortisone in the synovial cells (intracrinology), appear important in the pathophysiology of RA [6].

The RA inflammatory process therefore induces changes in synovial fluid composition, edema of synovial tissue, as well as redistribution of interstitial fluid, while sleeping contributes to clinical stiffness of the joints that is most pronounced in the early morning [7]. All of these processes are closely linked to regulatory interactions between the endocrine, nervous and immune systems, with distinct 24-hour daily rhythms (neuroendocrine immunology).

While the role of IL-6 and TNF alpha in the regulation of inflammatory and immune responses, particularly in RA, is well established, the increased production of other proinflammatory cytokines such as IL-8, IL-12 and IL-17 by primary and secondary immune cells may also be implicated in the circadian process [7]. Furthermore, in polymyalgia rheumatica (PMR), symptoms of pain and stiffness typically are most prominent during the early morning, similar to RA [2]. There are indications for circadian variation of TNF alpha and IL-6 secretion, with peak values in the early morning hours. Therefore, given the known high GC sensitivity of PMR, time-adapted GC therapy can be hypothesized to probably be more effective than the currently used standard regimen in improving clinical symptoms [8-10].

Of note, in ankylosing spondylitis - another inflammatory arthritic condition - pain and stiffness also seem to be most prominent during the early morning hours [2]. Finally, it is now also evident that symptoms of diseases such as RA, which is T helper 1 dependent, but also asthma, which is T helper 2 dependent, are influenced by diurnal rhythms and natural regulatory T cells [11]. In particular, secretion of IL-2, interferon gamma and IL-10 by naïve CD4^+ ^T cells follows a diurnal rhythm.

## Dysfunction of the HPA axis in RA and other chronic inflammatory diseases

From a GC perspective, the general problem of RA and other inflammatory diseases is that serum cortisol concentrations are inadequately low relative to inflammation [12,13]. What exactly does this mean?

### Inflammatory stress activates the HPA axis

In a fairly heroic study in 18 healthy young men, either saline or low or high doses of recombinant human IL-6 were infused into one femoral artery for 3 hours [14]. Subjects experienced clinical symptoms such as shivering and discomfort during high-dose IL-6 administration, but were asymptomatic during low-dose IL-6 administration. Plasma cortisol concentrations did not change during infusion of saline but markedly increased during both high and low doses of IL-6. While concentrations of plasma cortisol declined after 2 hours of infusion in low doses of IL-6, they remained elevated in high doses of IL-6 at 3 hours of infusion. During both IL-6 trials, plasma cortisol levels returned to preinfusion values after 3 hours of recovery [14].

**Figure 1:**
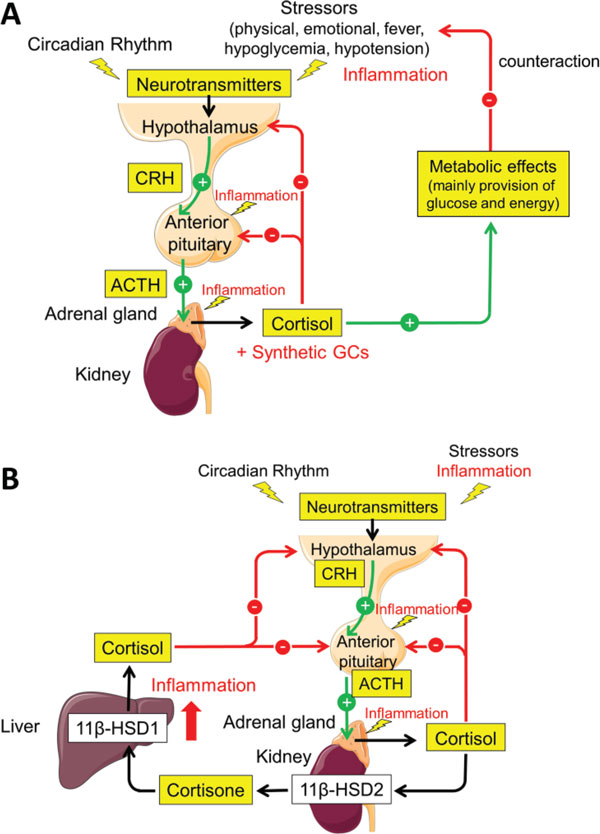
**Function and dysfunction of the hypothalamic-pituitary-adrenal axis in inflammation**. **(A) **Activation of the hypothalamic-pituitary-adrenal (HPA) axis and negative feedback regulation. The central circadian oscillator and different stressors (physical, emotional, fever, hypoglycemia, or hypotension) during physiological stress reactions trigger the hypothalamus to release corticotropin-releasing hormone (CRH). CRH acts on the anterior pituitary and induces release of adrenocorticotropic hormone (ACTH), which in turn stimulates the adrenal gland to produce and release cortisol. Cortisol exhibits its known metabolic effects (mainly provision of glucose and energy), which serve to counteract the stressor. Inflammation can also trigger the HPA axis. In the physiological regulation of the HPA axis, cortisol release is terminated by negative feedback regulation of cortisol on the hypothalamus and anterior pituitary. Synthetic glucocorticoids (GCs) applied in GC therapy can cause negative feedback regulation. This can lead to adrenal suppression. **(B) **A new concept for the feedback loop: the hepato-hypothalamic-pituitary-adrenal-renal axis. The HPA axis is extended by GC metabolism: cortisol is converted to cortisone mainly by the kidney, via 11β-hydroxysteroid dehydrogenase (11β-HSD) type 2, in order to protect the nonspecific mineralocorticoid receptor from activation by cortisol. The major organ for converting cortisone to cortisol is the liver, via 11β-HSD1. In chronic inflammation, conversion from cortisone to cortisol by 11β-HSD1 is increased (reviewed in [20]). This may amplify negative feedback and explain HPA dysfunction in inflammation. SERVIER medical art images were used for generation of figures.

The increase of cortisol levels in reaction to IL-6 infusion is provoked by activation of the HPA axis (Figure 1A). Remarkably, the relation between IL-6 levels and the adrenocorticotropic hormone (ACTH)/cortisol levels is linear. In a study of 15 healthy young men in which recombinant IL-6 was applied subcutaneously, plasma ACTH concentrations and plasma cortisol levels increased dose dependently, and the ratio of hormone to IL-6 serum levels was constant [15].

However, repeated IL-6 administration leads to adaptation of the HPA response. During a phase I trial, patients with cancer and a good performance status received daily morning subcutaneous injections of 30 μg/kg IL-6 for 7 consecutive days. IL-6 caused impressively marked and prolonged elevations of plasma ACTH and cortisol on the first day, but blunted ACTH responses on the seventh day of treatment, perhaps as a result of increased baseline cortisol levels [16].

### Inadequate cortisol secretion in relation to inflammation

In chronic inflammation, cortisol secretion appears to be inadequate in relation to inflammation. In a retrospective study with 34 patients with RA, 46 patients with reactive arthritis and 112 healthy subjects, the authors measured serum levels of IL-6, TNF and cortisol. The absolute levels of IL-6 were lower in healthy controls than in reactive arthritis and RA patients. However, the ratio of serum cortisol to serum cytokines was much higher in healthy controls than in reactive arthritis and RA patients, due to similar cortisol levels in all groups [13].

In a study by Crofford and colleagues comparing the circadian course of ACTH and cortisol levels in patients with RA and in healthy subjects, despite 10 times higher serum levels of cytokines in patients with RA, serum level curves of ACTH and cortisol were identical, with similar peaks in the early morning and lower second peaks in the afternoon [12]. The ACTH/cortisol hormone secretion in patients with RA is thus inadequate in relation to inflammation. This inadequacy is one reason - apart from their pharmacological effects - why low-dose GCs represent such an important part of modern RA therapy [17]. Data from the German registry show that GCs had been used in 55% of patients with RA in 1996 and were still used in 55% of patients with RA in 2010, even if the frequency of patients using low-dose therapy ≤7.5 mg prednisone equivalent increased from 40% to 48% [18].

Dysfunction of the HPA axis can also be found in PMR/ giant cell arteritis. In a study comparing serum values of ACTH, cortisol and CRP in patients with PMR/giant cell arteritis and controls, ACTH and cortisol levels were not different in patients with PMR/giant cell arteritis and controls, whereas the ratios of serum ACTH/serum CRP and serum cortisol/serum CRP were significantly lower in PMR/giant cell arteritis patients than in healthy controls [19]. Thus, in PMR/giant cell arteritis there also appears to be an inadequate cortisol secretion in relation to inflammation in terms of relative adrenal insufficiency. GC therapy can be regarded - at least in part - as supplemental GC therapy, supported by the high GC sensitivity in these diseases [4].

### New concept for the feedback loop: the hepato-hypothalamic-pituitary-adrenal-renal axis

How may the dysfunction of the HPA axis in chronic inflammation be induced? Recently, evidence has accumulated, been reviewed and presented as a concept that dysfunction of the HPA axis in chronic inflammation is not simply an adaptation to chronic stress, but may be due to increased negative feedback of active cortisol on the HPA axis [20].

The HPA axis has been recognized to be extendable to a hepato-hypothalamic-pituitary-adrenal-renal axis by GC metabolism. Active cortisol is converted to inactive cortisone mainly by the kidney, via 11β-hydroxysteroid dehydrogenase (11β-HSD) type 2, in order to protect the nonspecific mineralocorticoid receptor from activation by cortisol. On the other hand, the major organ for converting inactive cortisone to active cortisol is the liver, via 11β-HSD1 (Figure 1B).

Expression of 11β-HSD1 is markedly enhanced by TNF and proinflammatory cytokines [20]. The liver therefore becomes an important player in systemic inflammation, even if the conversion also occurs in multiple other tissues including the brain, adipocytes, vascular cells, osteoblasts and fibroblasts. Given the role of the 11β-HSD1 in GC metabolism, its effect on the HPA axis and its interaction with inflammatory cytokines, it is hypothesized that in chronic inflammatory diseases, cytokine-induced increased expression of 11β-HSD1 induces a change in the HPA axis [20]. Increased negative feedback of active cortisol on the HPA axis induced during inflammation may thus be the mechanism of dysfunction of the HPA axis in chronic inflammation (Figure 1B).

## Adrenal suppression by glucocorticoid therapy

### Tertiary adrenal insufficiency induced by glucocorticoid therapy

During the physiological regulation of the HPA axis, cortisol release is terminated by negative feedback regulation of cortisol on the hypothalamus and anterior pituitary (Figure 1A). Also, synthetic GCs - as applied in GC therapy - can cause negative feedback regulation, leading to adrenal suppression in terms of tertiary adrenal insufficiency [21].

Tertiary adrenal insufficiency typically becomes manifest in patients treated long term with synthetic GCs during situations of stress; for example, infections or operations [22]. Tertiary adrenal insufficiency generally has a less dramatic presentation than primary adrenal insufficiency; acute circulatory collapse seems rare, because aldosterone levels, which are controlled pre-dominantly by the renin-angiotensin system, are preserved [21]. GC-treated patients may be identified by symptoms of Cushing's syndrome [22].

### Diagnosis of tertiary adrenal insufficiency

The diagnosis of tertiary GC-induced adrenal insufficiency is based on the patient's history (therapy with GCs), low plasma cortisol and ACTH levels near zero, and functional tests such as the corticotropin-releasing hormone (CRH) or ACTH stimulation test. The CRH test allows testing of the adrenal partial function of the anterior pituitary. In principle, CRH injection induces cortisol secretion (via ACTH). In practice, 100 μg CRH are injected intravenously, and blood samples are acquired 0, 60, 90, and 120 minutes after injection for analysis of ACTH and cortisol plasma concentrations. The CRH test can be interpreted as normal with ACTH increase >50% and cortisol increase >5 μg/dl. In tertiary adrenal insufficiency (and adrenal Cushing's syndrome), no increase can be measured (whereas in central Cushing's syndrome, exaggerated increase can be found).

The ACTH test allows testing of primary and tertiary adrenal insufficiency. In the test, 250 μg ACTH are injected intravenously and blood samples are acquired 0, 60, and 120 minutes after injection for analysis of cortisol plasma concentrations. Increases of cortisol >10 μg/dl (276 nmol/l) would be normal [22].

### Factors that determine adrenal insufficiency

As is well known, occurrence of GC-induced tertiary adrenal insufficiency is dependent on different factors: individual sensitivity, GC dose, duration and preparation of GC therapy, and timing of application (circadian).

#### Individual sensitivity

The influence of individual factors on adrenal suppression is not yet well understood. In a study in RA patients and controls, 1.5 mg dexamethasone was given orally at 11:00 p.m., which was followed by application of CRH intravenously at 3:00 p.m. on the following day [23]. Normal controls and a number of the patients showed a suppression of ACTH and cortisol response as expected. However, three of seven patients (43%) with active RA (without GC therapy) did not exhibit the normal feedback control mechanism. This suggests that there might be a subpopulation of patients with RA who have impaired GC feedback [23].

In a study investigating the adrenal response after stopping GC treatment, a CRH test was performed in 75 patients who had received at least 25 mg prednisone daily for up to 30 days [24]. Interestingly, about one-half of the patients (34 of 75 patients) again showed a reduced response to CRH on the day after stopping treatment.

#### Glucocorticoid dose

The frequency of adrenal suppression increases with increasing GC dosages. In arthritis and asthma patients treated with prednisone equivalent doses ranging from 5 to 20 mg, cortisol response in the ACTH test was normal (that is, cortisol rise ≥7 μg/dl) in all of the patients taking a single morning dose of 5 to 7.5 mg prednisone, was blunted (that is, cortisol rise <7 μg/dl) in 33% and 47% of the patients taking 10 to 12.5 mg and 15 mg prednisone, respectively, and was suppressed (no rise) in 44% of the patients taking 20 mg prednisone [25].

Evidence that low-dose GC therapy can also result in adrenal suppression was found in a more recent randomized, double-blind and placebo-controlled trial in patients with RA investigating the plasma cortisol response in ACTH testing before and after 12 weeks of treatment with 7.5 mg prednisolone [26]. After 12 weeks of 7.5 mg prednisolone, the mean values for the 60-minute response to ACTH were reduced by 35%. Following treatment, 46% of patients taking 7.5 mg prednisolone failed to reach the normal maximum cortisol response to ACTH, even if the HPA axis response generally remained within the normal range [26].

#### Duration and preparation of glucocorticoid therapy

Abnormal diurnal rhythms of plasma cortisol in patients with RA were found to be related to the total dose of GC given and to the duration of therapy, but not to the mean daily dose or the daily regimen of therapy [27]. Patients receiving triamcinolone, dexamethasone and betamethasone had a higher prevalence of abnormal diurnal rhythm of plasma cortisol when compared with patients receiving prednisone or prednisolone, or methylprednisolone. The significance of this finding is uncertain since patients receiving the former group of drugs had received their GC therapy in higher dosage and for longer duration [27].

#### Circadian application

In the 1960s several studies confirmed that splitting the daily dose into several divided doses strongly increases the risk of adrenal suppression. For example, whereas endogenous cortisol secretion was not altered with a single dose of 8 mg triamcinolone given at 8:00 a.m., application of four divided 2 mg doses resulted in marked suppression of cortisol levels [28]. This is the reason why GC therapy in general is applied as a single daily dose.

The time point of application of the single daily dose also plays a role for adrenal suppression. This can be explained easily: circadian GC secretion exhibits two peaks, one large peak in the morning around 8:00 a.m. and a smaller peak in the afternoon around 2:00 p.m. [12]. Of note, cortisol levels are high during the first peak in the morning, causing downregulation of ACTH levels via negative feedback regulation. In consequence, cortisol secretion is also downregulated. At a certain point, reduced cortisol levels cause upregulation of ACTH again, leading in turn also to upregulation of cortisol secretion during the second peak in the afternoon. If exogenous GCs were applied in the evening, the so-called quiet period for the adrenal gland [29], this would cause a negative signal on ACTH and therefore also cortisol secretion in the morning.

This has been demonstrated in several experimental studies in healthy persons: 4-hour methylprednisolone infusion (0.7 mg/hour) between midnight and 4:00 a.m. induced severe adrenal suppression, whereas 4-hour infusion between 4:00 and 8:00 a.m. or between 4:00 and 8:00 p.m. induced moderate adrenal suppression. In contrast, 8-hour infusion (twice the methylprednisolone dose delivered in the other clock-hour trials) between 8:00 a.m. and 4:00 p.m. exerted no adrenal suppression [29,30]. Dexamethasone 0.5 mg administered at 8:00 a.m. or 4:00 p.m. caused only temporary suppression of cortisol secretion, whereas the same amount given at midnight produced virtually complete suppression of cortisol production for a full 24-hour period [31]. For this reason, GC therapy in general is applied in a single morning dose, as first recommended by Di Raimondo and Forsham in 1956 [32,33].

#### Dose splitting

This approach of choosing the right time for GC application primarily focused on chronotoxicological aspects; that is, rhythm-dependent differences in the manifestation and severity of adverse effects. However, despite the practice of the single morning dose, some patients receiving GC therapy for RA required a nocturnal GC dose to control morning stiffness [34]. This brought chronoeffectiveness into focus; that is, rhythm-dependent differences in the magnitude of the desired therapeutic effects [35].

The question is whether dose splitting into a morning and an evening dose is adequate for chronotherapy. There are reports showing no evidence of HPA suppression in any of the patients who received a low single daily dose of GC treatment, even when the dose was given at bedtime [25,36,37]. In contrast, dose splitting with morning and evening doses appears to cause more HPA suppression; in seven patients who were receiving prednisolone twice daily in a similar total daily dose as compared with these single daily dose experiments, three patients showed HPA suppression and two patients had lost growth hormone responsiveness [36]. However, splitting is necessary in some patients, so when is the best time for dose splitting? There is evidence from a French study using dutimelan 8-15 mite - a GC preparation with a morning (8:00 a.m., Dragee A: 3.5 mg prednisolone acetate + 2 mg prednisolone alcohol) and an afternoon (3:00 p.m., Dragee B: 1.5 mg prednisolone alcohol + 7.5 mg cortisone acetate) application - that the morning peak of endogenous cortisol secretion was preserved [38]. It can be concluded that, if dose splitting is necessary, exogenous GCs should be applied in the morning and early afternoon (3:00 p.m.) (2/3 + 1/3 dose).

## Does modified-release prednisone aggravate adrenal suppression?

### Chronotherapy with modified-release prednisone

From the pharmacokinetic standpoint there is no reason why prednisolone should be given at any particular time of day or night, as prednisolone pharmacokinetics does not show a diurnal rhythmicity [39]. However, treatment in the early afternoon or evening may be not be sufficient to dampen the response in the early morning, due to the short half-life of prednisolone (2 hours) and the intense immune activation during the night [1,39,40].

Several studies had suggested a greater effect of bedtime or night doses (2:00 a.m.) in comparison with morning doses of conventional prednisone on morning stiffness [34,41-43]. However, a regimen that requires regular waking of the patient at 2:00 a.m. was considered impractical for the therapeutic routine. Other studies had found no differences [44,45], and the influence on the HPA axis was not examined in these studies [34,41-45].

These considerations and observations led to the development of a new modified-release (MR) prednisone tablet formulation [46]. MR prednisone releases prednisone approximately 4 hours after ingestion; that is, at approximately 2:00 a.m. if taken at 10:00 p.m. bedtime. The CAPRA-1 study was a double-blind treatment with MR prednisone in comparison with morning administration of immediate-release prednisone for 12 weeks, followed by 9-month open-label extension treatment with MR prednisone (total duration of study 12 months) [46,47]. MR prednisone produced a clinically relevant reduction of morning stiffness of the joints in addition to all known therapeutic effects of immediate-release prednisone. In the open-label extension treatment, sustained reduction in morning stiffness was found in the MR prednisone-treated group.

### CAPRA-1 subgroup study with corticotropin-releasing hormone tests

These results lead to the question of whether chronotherapy with MR prednisone affects adrenal suppression. The influence of long-term, low-dose chronotherapy with MR prednisone on the HPA axis was investigated by CRH tests in a subgroup of 28 patients in the CAPRA-1 study [48]. The CRH tests were performed at baseline, at the end of the double-blind phase, and at the end of the 9-month open-label extension. Sixty-two valid tests were obtained in 28 patients.

There were no measurable differences in mean cortisol changes after CRH injection between baseline and the end of the study [48]. Furthermore, there was no indication that changing treatments from immediate-release prednisone to MR prednisone increased the risk of HPA axis insufficiency, or resulted in deterioration of preexisting suppression. Fifty percent of the patients showed a normal response (change ≥5 μg/dl) in the CRH test in immediate-release and MR prednisone treatment groups, 37.5% and 36.7%, respectively, of the patients showed suppressed response (change >0 to <5 μg/dl), and 12.5% and 13.3%, respectively, of the patients showed no response (no change). There was thus no difference between immediate-release prednisone and MR prednisone in numbers of normal/suppressed/no response reactions. In addition, no adverse events that could be attributed to HPA axis insufficiency were observed during treatment with low-dose MR prednisone for the entire treatment period of 12 months [48].

### Effect of modified-release prednisone on cortisol levels

A recent study showed an increase of endogenous cortisol after 2 weeks of MR prednisone therapy in patients with active RA who had received no GCs by any route in the preceding 3 months [49]. MR prednisone released at 2:00 a.m. suppressed the pathological early morning rise in plasma IL-6 in RA. The nocturnal rise in plasma cortisol was not suppressed but was enhanced with a peak value increase from 14.1 to 19.3 μg/dl, consistent with a changing relationship between HPA axis and immune system activation [49]. This observation may be an indication that the HPA axis is preserved and is activated even more during MR prednisone treatment compared with pre-MR prednisone treatment.

## Conclusions

From a GC perspective, circadian rhythms of the HPA axis and connected subsystems, including the immune system, appear to be essential for understanding of pathophysiology and treatment in rheumatology. The circadian rhythm of the HPA axis in chronic inflammatory diseases may be defective in terms of not bringing the body into a position to overcome the signs and symptoms of the disease. GC therapy serves as a necessary aid to overcome the disease and perhaps restore the deranged circadian rhythm. In a number of patients (around 50%), GC therapy causes adrenal suppression, probably mainly due to as yet undefined individual factors (apart from dose, substance and duration of therapy). In order not to aggravate adrenal suppression, GC therapy should be applied in accordance with the circadian rhythm, to achieve greatest efficacy along with highest safety. It has been suggested that when the single morning dose is not effective enough to achieve sufficient disease control, especially in patients with strong night symptoms and morning stiffness, split doses in the morning and afternoon, or chronotherapy with MR prednisone, can to some extent avoid aggravation of adrenal suppression.

## Abbreviations

ACTH: adrenocorticotropic hormone; CRH: corticotropin-releasing hormone; GC: glucocorticoid; HPA: hypothalamic-pituitary-adrenal; 11β-HSD: 11β-hydroxysteroid dehydrogenase; IL: interleukin; MR: modified release; PMR: polymyalgia rheumatica; RA: rheumatoid arthritis; TNF: tumor necrosis factor.

## Competing interests

CMS has received honoraria and travel expenses from Merck Serono and Mundipharma Int Ltd. RHS has received reimbursements, fees or funding from Horizon Pharma and Mundipharma Int Ltd. MC has received consultancy fees and honoraria from Horizon Pharma (formerly Nitec Pharma) and Mundipharma Int Ltd. FB has received consultancy fees, honoraria and travel expenses from Merck Serono, Horizon Pharma (formerly Nitec Pharma) and Mundipharma Int Ltd, and grant support from Merck Serono and Horizon Pharma.

## Authors' contributions

CMS and FB mainly contributed to the sections Dysfunction of the HPA axis in RA and other chronic inflammatory diseases, Adrenal suppression by glucocorticoid therapy, and CAPRA-1 subgroup study with corticotropin-releasing hormone tests. RHS mainly contributed to the sections New concept of the feedback loop: the hepato-hypothalamic-pituitary-adrenal-renal axis, and Effect of modified-release prednisone on cortisol levels. MC mainly contributed to the section Circadian rhythms in RA and other chronic inflammatory diseases.

## Declaration

This article has been published as part of *Arthritis Research & Therapy* Volume 16 Suppl 2, 2014: At the interface between immunology and endocrinology in rheumatic diseases. The full contents of the supplement are available at http://arthritis-research.com/supplements/16/S2.

This supplement was proposed, developed and commissioned by *Arthritis Research & Therapy *and was funded by an educational grant from Horizon Pharma Inc. All published articles were independently prepared by the authors and have undergone peer review in accordance with the journal's standard policies and processes. Horizon Pharma Inc had no input into the topics covered or the articles themselves. The Supplement Editor was appointed by the journal and declares that they have no competing interests. This article mentions MR Prednisone (Trade Name: Lodotra or Rayos) as a product of Horizon in the text (directly or indirectly) and accordingly cite the respective studies.
